# The end-tidal alveolar dead space fraction for risk stratification during the first week of invasive mechanical ventilation: an observational cohort study

**DOI:** 10.1186/s13054-023-04339-3

**Published:** 2023-02-09

**Authors:** Anoopindar K. Bhalla, Ariya Chau, Robinder G. Khemani, Christopher J. L. Newth

**Affiliations:** 1 Department of Anesthesiology and Critical Care Medicine, Children’s Hospital Los Angeles, Department of Pediatrics, Keck School of Medicine, University of Southern California, 4650 Sunset Blvd, MS#12, Los Angeles, CA 90027 USA; 2 Division of Cardiology, Department of Pediatrics, Lucile Packard Children’s Hospital at Stanford, Stanford University School of Medicine, Palo Alto, CA USA

**Keywords:** Ventilators, Mechanical, Respiratory distress syndrome, Mortality, Pediatrics, Critical care outcomes

## Abstract

**Background:**

The end-tidal alveolar dead space fraction (AVDSf = [PaCO_2_−P_ET_CO_2_]/PaCO_2_) is a metric used to estimate alveolar dead space. Higher AVDSf on the first day of mechanical ventilation is associated with mortality and fewer ventilator-free days. It is not clear if AVDSf is associated with length of ventilation in survivors, how AVDSf performs for risk stratification beyond the first day of ventilation, or whether AVDSf adds predictive value to oxygenation (oxygenation index [OI]) or severity of illness (Pediatric Risk of Mortality [PRISM III]) markers.

**Methods:**

Retrospective single-center observational cohort study of children and young adults receiving invasive mechanical ventilation. In those with arterial or capillary blood gases, AVDSf was calculated at the time of every blood gas for the first week of mechanical ventilation.

**Results:**

There were 2335 children and young adults (median age 5.8 years [IQR 1.2, 13.2]) enrolled with 8004 analyzed AVDSf values. Higher AVDSf was associated with mortality and longer length of ventilation in survivors throughout the first week of ventilation after controlling for OI and PRISM III. Higher OI was not associated with increased mortality until ≥ 48 h of ventilation after controlling for AVDSf and PRISM III. When using standardized variables, AVDSf effect estimates were generally higher than OI for mortality, whereas OI effect estimates were generally higher than AVDSf for the length of ventilation in survivors. An AVDSf > 0.3 was associated with a higher mortality than an AVDSf < 0.2 within each pediatric acute respiratory distress syndrome severity category.

The maximum AVDSf within 12 h of intensive care unit admission demonstrated good risk stratification for mortality (AUC 0.768 [95% CI 0.732, 0.803]). AVDSf did not improve mortality risk stratification when added to PRISM III but did improve mortality risk stratification when added to the gas exchange components of PRISM III (minimum 12-h PaO_2_ and maximum 12-h PCO_2_) (*p* < 0.00001).

**Conclusions:**

AVDSf is associated with mortality and length of ventilation in survivors throughout the first week of invasive mechanical ventilation. Some analyses suggest AVDSf may better stratify mortality risk than OI, whereas OI may better stratify risk for prolonged ventilation in survivors than AVDSf.

**Supplementary Information:**

The online version contains supplementary material available at 10.1186/s13054-023-04339-3.

## Background

Alveolar dead space, alveoli that receive ventilation without perfusion, is common in critically ill patients receiving mechanical ventilation [1–3]. Primary etiologies for increased alveolar dead space include alveolar overdistension, pulmonary vascular dysfunction or thrombosis, and low cardiac output. An estimate of alveolar dead space is the end-tidal alveolar dead space fraction (AVDSf = [PaCO_2_−P_ET_CO_2_]/PaCO_2_) [4]. Several studies have demonstrated the prognostic value of elevated AVDSf in the first 24 h of mechanical ventilation in children with acute hypoxemic respiratory failure and in general cohorts of critically ill mechanically ventilated children [1, 5, 6]. An association between dead space metrics and mortality is also consistently found in adult critically ill patients [7–10].

In children with parenchymal lung disease, oxygenation and alveolar dead space abnormalities often occur together with each providing discrete data on ventilation and perfusion in the diseased lung [11]. Although there is conflicting evidence, there is some research to suggest that AVDSf may have a stronger association with mortality than oxygenation markers early in pediatric acute hypoxemic respiratory failure [5, 6, 11]. While markers of dead space have been considered for both adult and pediatric definitions of acute respiratory distress syndrome (ARDS), they have not been included in these definitions for several reasons [12, 13]. These may relate to the infrequent use of capnography in adults and neonates and imprecise surrogate markers for dead space (such as corrected minute ventilation or ventilatory ratio) without capnography. Furthermore, there are limited data on the prognostic value of AVDSf beyond the initial 24 h of invasive mechanical ventilation and whether AVDSf adds predictive value over readily available oxygenation markers. Notably, there are no data on whether AVDSf improves the predictive ability of current pediatric critical care severity of illness scores.

We sought to determine, in a large general cohort of critically ill mechanically ventilated children and young adults with a broad range of primary diagnoses, the association between AVDSf and (1) mortality and (2) length of ventilation in survivors over the first week of invasive mechanical ventilation and evaluate whether AVDSf provides additive predictive value over oxygenation markers and severity of illness scores. We hypothesized that AVDSf is a strong prognostic marker for mortality and prolonged ventilation in survivors independent of the severity of oxygenation abnormality or severity of illness.

## Methods

This is a retrospective observational cohort study of children and young adults who received invasive mechanical ventilation for any reason in the Children’s Hospital Los Angeles (CHLA) Pediatric Intensive Care Unit (PICU) and were monitored with time-based capnography between August 2012 and September 2021. Children with no arterial or capillary blood gas values within the first 7 days of invasive mechanical ventilation were excluded. Children excluded for this reason generally had central venous access and were monitored with venous blood gases. Venous PCO_2_ is not well accepted as an accurate estimate of PaCO_2_, particularly in patients with hemodynamic instability; therefore, these patients were excluded [14]. Time-based capnography is part of standard monitoring for children on conventional ventilation in the CHLA PICU. There were no age-based exclusion criteria; however, neonates and young adults older than 21 years of age are rarely admitted to the CHLA PICU. Children with cyanotic heart disease are admitted to a separate cardiothoracic ICU at CHLA. The CHLA institutional review board approved this study with a waiver of informed consent (CHLA 22-00050).

### Data extraction

Continuous data from the bedside monitor at the time of each arterial or capillary blood gas, which were obtained for clinical purposes, were averaged during the 1 min before and after the blood gas. Monitor data included P_ET_CO_2_ and SpO_2_. For a subgroup of patients, continuous data on ventilator parameters including mean airway pressure (MAP) and FiO_2_ were also available. Demographic, diagnostic, severity of illness (Pediatric Risk of Mortality [PRISM III]), and blood gas data were obtained from the electronic medical record and an administrative database [15].

### Variable definition

Alveolar dead space was quantified with the end-tidal alveolar dead space fraction (AVDSf = [PaCO_2−_P_ET_CO_2_]/PaCO_2_) at the time of each blood gas during the first week of invasive mechanical ventilation [4]. An arterial blood gas (PaCO_2_) was used preferentially, but a capillary PCO_2_ could be substituted for AVDSf calculation when an arterial blood gas was not available. If the calculation of AVDSf resulted in a negative value, a zero was imputed for analysis. We have previously demonstrated that AVDSf is a reasonable surrogate for alveolar dead space calculated from volumetric capnography [4]. However, AVDSf, similar to most commonly used methods of dead space estimation, is influenced by the degree of intrapulmonary shunt [16, 17]. Oxygenation abnormality was quantified at the time of each blood gas with oxygenation index (OI = [MAP*FiO_2_*100]/PaO_2_) and, when MAP was unavailable, PF ratio (PaO_2_/FiO_2_) (if FiO_2_ was available). Ventilator data were obtained through a continuous data feed to the monitor, and when this data feed was not set up or disconnected, some variables were not available. When a capillary blood gas was used, PaO_2_ was estimated from SpO_2_, using the reported formula by Sauthier et al. to calculate OI and PF ratio [18].

Time windows were created for blood gases from the time of initiation of invasive ventilation (intubation [0–6 h], 12 h [6–18 h], 24 h [18–36 h], 48 h [36–60 h], 72 h [60–84 h], 96 h [84–120 h] and 144 h [120–168 h]) with the closest blood gas to the time point of interest, which was within the time window, identified for the analysis. Pediatric acute respiratory distress syndrome (PARDS) severity was determined using OI based on Pediatric Acute Lung Injury Consensus Conference (PALICC) criteria [19]. When OI was unavailable (missing MAP), ARDS severity was determined with Berlin criteria (using PF ratio) [12]. The first AVDSf within each PARDS severity category for each patient (if the patient had at least one AVDSf within a given PARDS severity category) was identified for analysis of mortality by AVDSf. AVDSf was categorized for analyses using the 75th and 90th percentile in children with a nonzero AVDSf (< 75th percentile: AVDSf < 0.2, 75th to 90th percentile: AVDSf ≥ 0.2 and < 0.3, ≥ 90th percentile: AVDSf ≥ 0.3) [1]. The natural log of PRISM III probability of death (POD) was used in modeling.

### Outcomes

Our primary outcomes were PICU mortality and length of invasive mechanical ventilation in survivors. Multiple courses of invasive mechanical ventilation were summed to determine the total length of invasive mechanical ventilation if the time interval between courses was < 24 h.

### Statistical analyses

We first performed descriptive analyses to determine the association between AVDSf categories and demographics, ventilator settings, blood gas data, and outcomes. AVDSf between survivors and non-survivors at every time point was compared with a Mann–Whitney U test. Differences in mortality by AVDSf category within each PARDS severity category were compared with chi-square tests. A Bonferroni correction was used to adjust for multiple comparisons. In children with OI available, multivariable logistic regression models were constructed to determine the association between AVDSf and mortality at each time interval adjusting for OI and severity of illness (PRISM III). OI and PRISM III were a priori chosen as likely confounders based on previous research and biological plausibility. Similarly, cox regression models at each time point of interest were constructed for the association between AVDSf and length of ventilation in survivors (modeled as time to liberation from invasive mechanical ventilation). Age, categorized by quartiles, and primary diagnosis were evaluated as potential effect modifiers in multivariable modeling using interaction terms. An interaction with a p value < 0.2 was considered significant. Nested models (1) AVDSf and PRISM III or (2) OI and PRISM III were compared to the full model with all three variables (AVDSf, PRISM III, and OI) using likelihood ratio tests and change in AIC (Akaike information criterion) and BIC (Bayesian information criterion) at each time point. To quantify the association between each variable (AVDSf and OI) and outcome, each variable was standardized to their standard deviation and these values were also used in modeling. The area under the receiver operating curve (AUC) estimates for logistic regression models using (1) maximum 12-h AVDSf, (2) PRISM III, and (3) minimum 12-h PaO_2_ and maximum 12-h PCO_2_ (the gas exchange components of PRISM III) for the outcome of mortality were determined. The AUC estimates for the latter two models were compared to models when a maximum of 12-h AVDSf was added as a covariate using the STATA roccomp command. All analyses were performed with STATA 17 (StataCorp LLC).

## Results

There were 14,299 children and young adults admitted during the study time period with 4163 receiving invasive mechanical ventilation. Of these children and young adults, 2335 had one or more arterial or capillary blood gas and corresponding capnography data within the first 7 days of invasive mechanical ventilation. There were 1920 children and young adults with simultaneous MAP and FiO_2_ data to calculate at least 1 OI in the first 7 days of invasive mechanical ventilation. The analysis included 8004 AVDSf values, 2741 (34.2%) using a capillary PCO_2_, with a median of 3 (IQR 1, 5) AVDSf values used per patient.

**Fig. 1:**
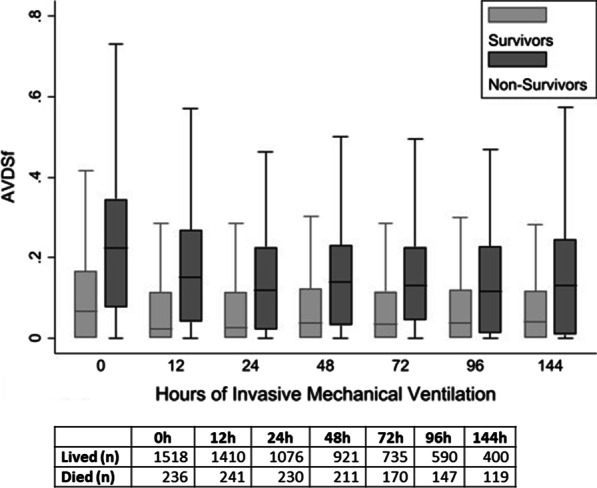
AVDSf by mortality over the first week of ventilation. Median AVDSf was significantly higher at all time points (all p = 0.0001). Box represents interquartile range with median at the center line. Whiskers are non-outlier range. Outlier values are not displayed

**Fig. 2:**
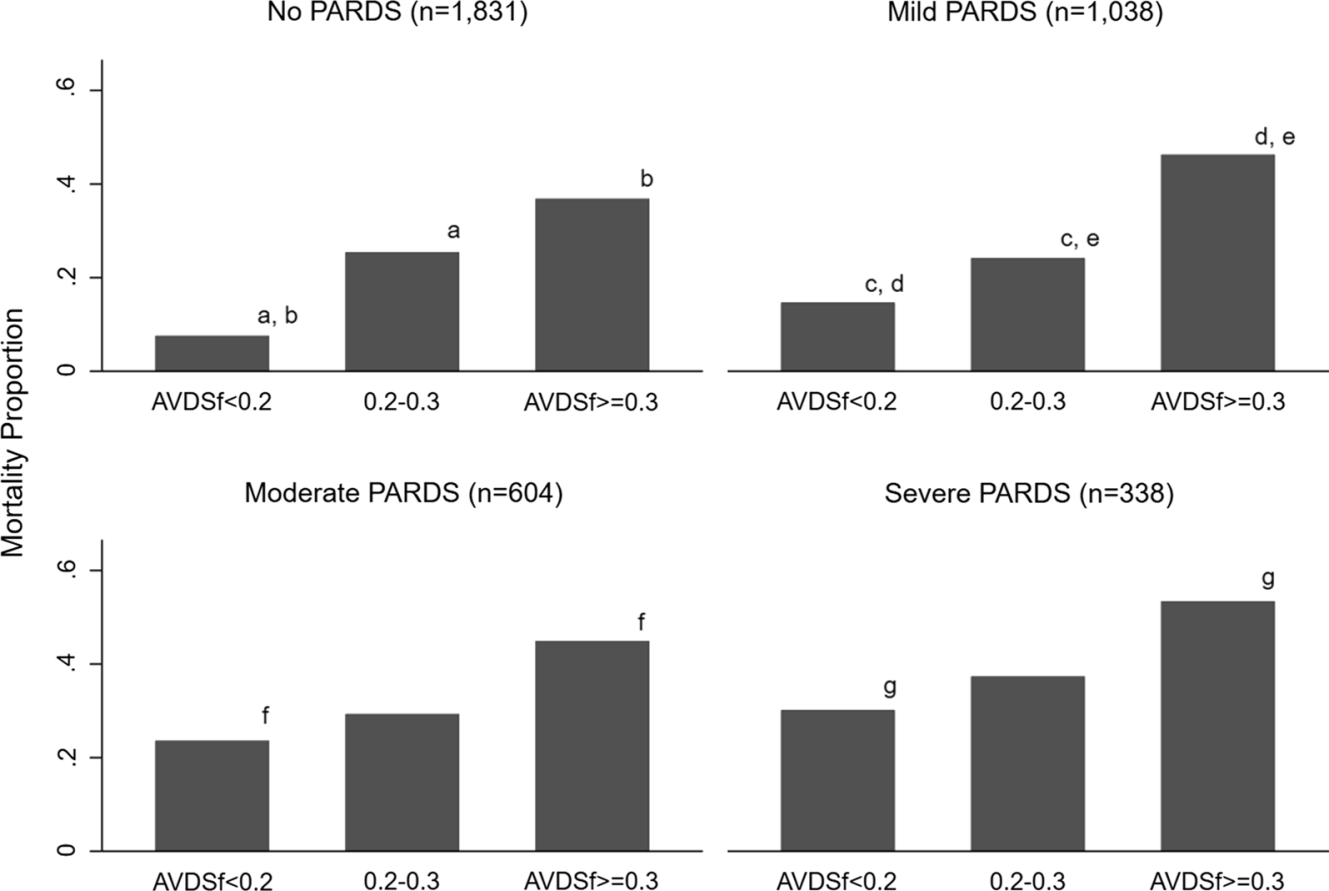
Mortality by AVDSf category within each PARDS severity category. For mortality comparisons within each PARDS category, a p value of 0.0167 was considered significant using a Bonferroni correction. Significant differences between categories are represented by lower case letters. The first AVDSf within each PARDS category for each patient was used in the analysis. Some patients are represented multiple times across PARDS categories. There are no patients represented multiple times within a PARDS category

When categorizing AVDSf, 1834 (78.5%) of values were < 0.2, 274 (11.7%) were between 0.2 and 0.3, and 227 (9.7%) were ≥ 0.3. In general, AVDSf was more likely to be elevated in children with primary respiratory or cardiovascular diagnoses, and higher AVDSf was associated with pH, PCO_2_, FiO_2_, positive end-expiratory pressure (PEEP), MAP, OI, and severity of illness (PRISM III)(all *p* < 0.0001) (Table 1). At every time point of interest during the first week of invasive mechanical ventilation, AVDSf was higher in non-survivors than survivors (all *p* = 0.0001) (Fig. 1). When stratifying by PARDS severity (no PARDS, mild PARDS, moderate PARDS, or severe PARDS), the risk of mortality increased in a near stepwise fashion as a function of AVDSf (all *p* < 0.01) and an AVDSf ≥ 0.3 was associated with a significantly higher mortality than an AVDSf < 0.2 within each PARDS severity category (Fig. 2, Additional file 1: Table S1).

**Table 1:** Patient, blood gas, and ventilation characteristics

	AVDSf < 0.2 (*n* = 1834)	AVDSf ≥ 0.2–0.3 (*n* = 274)	AVDSf ≥ 0.3 (*n* = 227)	*p* value
*Patient demographics*				
Age (years)	5.8 (1.3, 13.1)	6.4 (1.1, 13.9)	5.2 (0.8, 13.8)	0.67
Male	1029 (56.1%)	150 (54.7%)	139 (61.2%)	0.28
Primary diagnosis				< 0.0001
Neurologic	458 (25.0%)	41 (15.0%)	28 (12.3%)	
Respiratory	515 (28.1%)	111 (40.5%)	100 (44.1%)	
Cardiovascular	173 (9.4%)	53 (19.3%)	51 (22.5%)	
Gastrointestinal/Renal	231 (12.6%)	23 (8.4%)	10 (4.4%)	
Hematologic/Oncologic	64 (3.5%)	12 (4.4%)	8 (3.5%)	
Endocrinologic	9 (0.5%)	4 (1.5%)	4 (1.8%)	
Trauma	83 (4.5%)	12 (4.4%)	18 (7.9%)	
Other-Surgical	240 (13.1%)	9 (3.3%)	1 (0.4%)	
Other-Non Surgical	61 (3.3%)	9 (3.3%)	7 (3.1%)	
*Blood gas and ventilator data for first available AVDSf*				
Time from Initiation of Invasive Mechanical Ventilation (d)	0.11 (0.04, 0.39)	0.09 (0.04, 0.23)	0.08 (0.04, 0.20)	0.0097
Time from PICU Admission (d)	0.15 (0.03, 0.63)	0.16 (0.05, 0.71)	0.13 (0.03, 0.20)	0.36
pH	7.38 (7.33, 7.43)	7.32 (7.25, 7.38)	7.24 (7.13, 7.31)	0.0001
PaCO_2_	39 (35, 44)	46 (38, 55)	53 (43, 65)	0.0001
FiO_2_	0.40 (0.30, 0.41)	0.50 (0.40, 0.75)	0.62 (0.45, 1)	0.0001
V_T_/kg (ABW)	7.7 (6.1, 9.4)	7.0 (5.4, 8.9)	7.3 (5.3, 9.1)	0.009
MAP	10 (8, 12)	13 (10, 18)	15 (11, 18)	0.0001
PEEP	5 (5, 7)	7 (5, 10)	8 (6, 12)	0.0001
Oxygenation Index^a^	2.7 (1.9, 4.5)	7.0 (3.5, 15.6)	9.1 (4.6, 20.4)	0.0001
*Severity of illness and outcomes*				
PRISM III Score	4 (2, 8)	9 (4, 15)	12 (5, 22)	0.0001
PICU Length of stay in survivors (d)	5.2 (2.7, 10.5)	8.2 (3.8, 14.8)	9.6 (4.7, 18.1)	0.0001
Length of ventilation in survivors (d)	2.8 (0.9, 6.1)	4.9 (2.3, 10.4)	6.1 (2.7, 10.8)	0.0001
PICU mortality	150 (8.2%)	67 (24.5%)	88 (38.8%)	< 0.0001

FiO_2_, V_T_/kg, MAP, PEEP, and Oxygenation Index are reported for children when available. There were a total of 1758 children with FiO_2_, 1687 with V_T_/kg, 1638 with MAP, 1790 with PEEP, and 1623 with Oxygenation Index at the time of their first available AVDSf

*ABW* actual body weight; *PICU* pediatric intensive care unit; *V*_*T*_ tidal volume (exhaled)

### Mortality models

In multivariable models for all time points of interest, higher AVDSf was associated with mortality after controlling for OI and PRISM III (all *p* < 0.05 except 48-h [*p* = 0.087]) (Table 2). OI was not independently associated with increased mortality in multivariable modeling before the 48-h time window. At the time of intubation, adding OI to a model with AVDSf and PRISM III for the outcome of mortality was significant, but a higher OI was associated with lower mortality (OR 0.97 [0.95, 0.99], *p* = 0.043). Model performance improved at most time points when adding AVDSf to a model with OI and PRISM III (Additional file 1: Table S2). However, model performance did not improve when adding OI to a model with AVDSf and PRISM III before 48 h for the outcome of mortality (Additional file 1: Table S2). Using standardized variables, the effect size of AVDSf was higher than OI prior to 48 h and then generally similar to OI over subsequent time points for mortality models (Additional file 1: Table S3).

**Table 2:** Association between AVDSf and mortality

Model time window	*n*	AVDSf (per 0.1 increase)		Oxygenation index (per 1 increase)		PRISM III	
		OR (95% CI)	*p* value	OR (95% CI)	*p* value	OR (95% CI)	*p* value
Intubation (0–6 h)	1181	1.21 (1.03, 1.42)	0.018	0.97 (0.95, 0.999)	0.043	2.81 (2.41, 3.27)	< 0.0001
12 h (6–18 h)	1239	1.40 (1.16, 1.69)	< 0.0001	0.99 (0.96, 1.01)	0.19	2.56 (2.24, 2.93)	< 0.0001
24 h (18–36 h)	1020	1.24 (1.04, 1.49)	0.017	1.01 (0.99, 1.04)	0.27	2.31 (2.03, 2.62)	< 0.0001
48 h (36–60 h)	882	1.19 (0.98, 1.45)	0.087	1.04 (1.01, 1.07)	0.023	2.29 (2.00, 2.63)	< 0.0001
72 h (60–84 h)	689	1.47 (1.17, 1.84)	0.001	1.06 (1.02, 1.10)	0.003	2.30 (1.96, 2.70)	< 0.0001
96 h (84–120 h)	547	1.28 (1.03, 1.60)	0.026	1.06 (1.02, 1.10)	0.003	1.96 (1.68, 2.29)	< 0.0001
144 h (120–168 h)	399	1.64 (1.25, 2.15)	< 0.0001	1.07 (1.03, 1.13)	0.002	1.83 (1.53, 2.18)	< 0.0001

Multivariable logistic regression models were constructed for each time window within the first 7 days of invasive mechanical ventilation using the identified AVDSf, Oxygenation Index, and PRISM III for that time window

Age and primary diagnosis modified the relationship between AVDSf and mortality in all models (Additional file 1: Tables S4 and S5). However, there was no clear trend for any subgroup where the association between higher AVDSf and mortality was not present. The magnitude of the association and the statistical significance of the associations did differ by subgroup, with older children (> 1.2 years of age) and those with the cardiovascular disease having a stronger association between AVDSf and mortality.

In the subgroup of children with AVDSf values within 12 h of PICU admission (*n* = 1602), the maximum 12-h AVDSf demonstrated good risk stratification for mortality (AUC 0.768 [95% CI 0.732, 0.803]). PRISM III had an AUC of 0.925 (95% CI 0.908, 0.943) for mortality with no significant change when maximum 12-h AVDSf was added to the model (AUC 0.926 [95% CI 0.909, 0.943], *p* = 0.40). When evaluating minimum 12-h PaO_2_ and maximum 12-h PCO_2_, the primary gas exchange components of PRISM III, the AUC for mortality was 0.670 (95% CI 0.627, 0.712) and the addition of AVDSf to the model significantly improved the AUC to 0.775 (95% CI 0.740, 0.810)(*p* < 0.00001).

### Length of ventilation models

In multivariable models for each time point of interest, higher AVDSf was associated with a longer length of mechanical ventilation in survivors after controlling for OI and PRISM III (all *p* < 0.05 except at intubation [*p* = 0.076], 72 h [*p* = 0.28], and 144 h [*p* = 0.18]) (Table 3). Higher OI was independently associated with longer length of mechanical ventilation in survivors at all time points (all *p* < 0.05). Model performance improved at most time points for the length of ventilation in survivors when adding AVDSf to a model with OI and PRISM III (Additional file 1: Table S6). Using standardized variables, OI had a larger effect size than AVDSf on the length of mechanical ventilation in survivors at almost all time points (Additional file 1: Table S7).

**Table 3:** Association between AVDSf and time to extubation in survivors

Model time window	*n*	AVDSf (per 0.1 unit increase)		Oxygenation index (per 1 unit increase)		PRISM III	
		HR (95% CI)	*p* value	HR (95% CI)	*p* value	HR (95% CI)	*p* value
Intubation (0–6 h)	1028	0.95 (0.89, 1.01)	0.076	0.97 (0.96, 0.99)	< 0.0001	0.89 (0.85, 0.93)	< 0.0001
12 h (6–18 h)	1066	0.83 (0.77, 0.89)	< 0.0001	0.97 (0.96, 0.99)	< 0.0001	0.94 (0.90, 0.98)	0.002
24 h (18–36 h)	842	0.90 (0.83, 0.97)	0.009	0.97 (0.96, 0.99)	< 0.0001	0.91 (0.87, 0.95)	< 0.0001
48 h (36–60 h)	725	0.88 (0.81, 0.95)	0.002	0.96 (0.94, 0.98)	< 0.0001	0.97 (0.92, 1.02)	0.21
72 h (60–84 h)	567	0.95 (0.86, 1.05)	0.28	0.95 (0.93, 0.98)	< 0.0001	0.92 (0.87, 0.97)	0.002
96 h (84–120 h)	442	0.86 (0.77, 0.97)	0.01	0.97 (0.95, 0.99)	0.003	0.98 (0.93, 1.04)	0.51
144 h (120–168 h)	308	0.91 (0.78, 1.05)	0.18	0.96 (0.93, 0.99)	0.003	0.99 (0.93, 1.05)	0.75

Multivariable cox regression models were constructed for each time window within the first 7 days of invasive mechanical ventilation using the identified AVDSf, Oxygenation Index, and PRISM III for that time window

Age and primary diagnosis modified the relationship between AVDSf and length of ventilation in survivors in most models (Additional file 1: Tables S8, S9). However, there was no clear trend for any subgroup where the association between higher AVDSf and longer length of ventilation was not present. The magnitude and statistical significance of the associations did differ by subgroup, with older children (> 1.2 years of age) in general having a stronger association between AVDSf and length of ventilation in survivors.

In sensitivity analyses, limited to children with arterial blood gases, multivariable modeling results were similar (Additional file 1: Tables S10, S11). In these models, OI was not significantly associated with increased mortality until 96 h and AVDSf was not significantly associated with longer length of ventilation in survivors at intubation.

## Discussion

Our analyses confirm and expand on previous research demonstrating the strong association between higher AVDSf and mortality and prolonged length of mechanical ventilation in critically ill children. We have demonstrated that AVDSf is an important prognostic marker throughout the first week of invasive mechanical ventilation, beyond the previously reported range of the first 24 h. AVDSf provides additional risk stratification information independent of the severity of oxygenation abnormality or severity of illness at most time points in the first week of mechanical ventilation. In fact, early in the course of mechanical ventilation, higher OI is not associated with mortality after controlling for AVDSf. Previous reports of the association between AVDSf and length of ventilation have been limited to analyses of ventilator-free days which is a composite outcome of both length of ventilation in survivors and mortality. We have now demonstrated the independent association between AVDSf and each outcome (mortality and length of mechanical ventilation in survivors). Lastly, when considering a common pediatric severity of illness score (PRISM III), AVDSf improves risk discrimination for mortality when added to the current gas exchange components of PRISM III (maximum PaCO_2_ and minimum PaO_2_).

During the first hours of mechanical ventilation, patient physiology may change rapidly. In children with PARDS, ventilator management is aimed to achieve lung recruitment and decrease intrapulmonary shunt, thereby improving oxygenation. This likely contributes to the noted stronger association of AVDSf with mortality, in comparison with OI, early in mechanical ventilation that then equalizes over time. Poor oxygenation attributable to atelectasis or pulmonary edema may be more easily improved with positive pressure ventilation and reflect lower disease severity, than true alveolar consolidation. Previous studies have also demonstrated that OI may not perform as well as AVDSf for mortality risk stratification early in ARDS [5]. Nevertheless, no current diagnostic methods for ARDS or severity of illness scoring, in adults and children, include dead space metrics and most rely entirely on oxygenation metrics to assess the severity of pulmonary disease. Identification of patients at the highest risk for mortality early in the course of critical illness is crucial for weighing the risk–benefit ratio of potentially harmful interventions, such as neuromuscular blockade, and for timely enrollment in clinical trials to study new interventions. We have demonstrated in comprehensive analyses that across PARDS severity categories, ages, and primary diagnoses, AVDSf provides additional risk stratification information to oxygenation markers. Our results add to the accumulating evidence suggesting that dead space markers should be further considered for diagnostic severity scoring in ARDS and potentially for other severity of illness scores in both children and adults.

However, it is important to note that this analysis represents a subgroup of critically ill children, those that require conventional mechanical ventilation and are monitored with either arterial or capillary blood gases. This limits the generalizability of our results to critically ill children who do not meet these criteria. Capnography cannot be performed reliably when patients are not on respiratory support or in those supported with either noninvasive or non-conventional ventilation. Currently, there are also no validated methods for dead space calculation that do not require blood gas monitoring. While arterial blood gas monitoring practices vary between institutions, most critically ill children, even those with PARDS, are not monitored with arterial blood gases [19, 20]. In critically ill adults, the ventilatory ratio has been suggested as a method to estimate dead space as this method does not require capnography data [7]. However, the ventilatory ratio continues to require arterial blood gases monitoring and has not performed well in comparison to AVDSf in children [5]. Capillary PCO_2_ is likely an acceptable estimate of PaCO_2_, and the exclusion of capillary blood gases did not significantly alter our results in sensitivity analyses [21]. However, frequent monitoring of capillary blood gases may not be acceptable to patients and families. Other noninvasive methods of dead space assessment, including volumetric capnography curve analysis, have been proposed, but these have not been validated in children [22]. Additionally, most methods for dead space estimation such as AVDSf, physiologic dead space from the Bohr–Enghoff equation, or ventilatory ratio rely on the assumption that arterial PCO_2_ equals alveolar PCO_2_ and therefore may be influenced by intrapulmonary shunt [23, 24]. The development of methods to monitor dead space that overcomes these issues should be pursued to determine if dead space metrics may add prognostic information in all critically ill children.

It is often assumed that the strongest predictors of mortality are also most strongly associated with the length of ventilation. This forms the rationale for the common critical care outcome of ventilator-free days. However, as our data have demonstrated, this may not always be the case. AVDSf had a larger estimated effect size on mortality in the first 24 h of mechanical ventilation and continued to be significantly associated with mortality throughout the first week of ventilation. When considering that most children with PARDS die from multisystem organ failure, the potential importance of vascular dysfunction is highlighted [19]. Dead space reflects pulmonary perfusion abnormality and is more likely to reflect vascular dysfunction than oxygenation markers. On the other hand, at most time points, OI had a significantly larger estimated effect on the length of mechanical ventilation in survivors than AVDSf. The reasons for prolonged mechanical ventilation are numerous but mechanical ventilation liberation practices and spontaneous breathing trial eligibility are often reliant on oxygenation metrics (MAP or PEEP, FiO_2_, SpO_2_) [25, 26]. Therefore, it may not be surprising that OI performed better than AVDSf for risk discrimination of length of ventilation in survivors. It is crucial that these relationships are further studied and considered when choosing the appropriate patients and outcomes for clinical trials based on the expected mechanism of the effect of the intervention.

AVDSf did not add significantly to the performance of PRISM III for mortality prediction demonstrating that the prognostic data reflected by elevated AVDSf are captured in the variables considered in PRISM III. However, PRISM III is a cumbersome score that uses numerous data points and is rarely used clinically. AVDSf did significantly outperform PaCO_2_ and PaO_2_, the gas exchange metrics of PRISM III. Risk prediction, in various formats, in critically ill adults and children is the focus of a large existing, and growing, body of research. However, although evidence is accumulating on the prognostic importance of the easily calculated marker of dead space, almost no studies have considered dead space in predictive modeling. This is an area ripe for further research and consideration of dead space may improve risk stratification in diagnostic criteria, such as those for PARDS, and for the severity of illness scores.

The large cohort and closely matched physiologic values using continuous monitoring data are clear unique strengths of this study. The primary limitation of this study is obtaining data from a single center. It is possible that some site-specific practices, criteria for arterial line catheter placement, use of inhaled nitric oxide, or ventilator liberation for example, may lead to less applicability at other centers. However, the study results are consistent with numerous other studies demonstrating the prognostic relevance of dead space markers seemingly making this possibility less likely. We did not have the continuous ventilator data for OI calculation (MAP or FiO_2_ missing) for some patients. In patients with FiO_2_, we used Berlin Criteria for PARDS severity stratification but excluded them from multivariable modeling. It is possible PARDS severity was misclassified in some of these patients or that the relationship between AVDSf and outcome is different in the patients excluded from modeling. Our findings may require validation in multicenter observational studies.

## Conclusions

In conclusion, AVDSf is an important marker of the severity of illness associated with both mortality and length of ventilation in survivors, initially and over the first week of invasive mechanical ventilation for critically ill children. Some analyses suggest AVDSf performs better than OI for risk stratification of mortality, whereas OI performs better for risk stratification of the length of ventilation in survivors. AVDSf may be useful for additional mortality risk stratification within PARDS severity categories.

## Supplementary Information

**Additional file 1.** Supplement Tables 1–11.

## Data Availability

The data used for this study analysis are available from the corresponding author given there is a reasonable request for the data.

## Abbreviations

AIC: Akaike information criterion
AVDSf: End-tidal alveolar dead space fraction
BIC: Bayesian information criterion
FiO_2_: Fractional inspired oxygen
MAP: Mean airway pressure
OI: Oxygenation index
PARDS: Pediatric acute respiratory distress syndrome
PCO_2_: Partial pressure of carbon dioxide
PEEP: Positive end-expiratory pressure
PRISM: Pediatric risk of mortality
SpO_2_: Oxygen saturation (pulse oximetry)
