# Supplementary material for: The end-tidal alveolar dead space fraction for risk stratification during the first week of invasive mechanical ventilation: an observational cohort study
Source: Crit Care. 2023 Feb 9;27:54. doi: 10.1186/s13054-023-04339-3 (PMC9912669; doi:10.1186/s13054-023-04339-3)
Supplement: Supplementary file 1 — Additional file 1. Supplement Tables 1–11. [file 13054_2023_4339_MOESM1_ESM.docx]

**Title:** The End-Tidal Alveolar Dead Space Fraction for Risk Stratification during the First Week of Invasive Mechanical Ventilation: An observational cohort study

**SUPPLEMENT**

**Supplement Table 1:** Mortality by AVDSf category within each PARDS severity category

|  | No PARDS (n=1,831) | Mild PARDS (n=1,038) | Moderate PARDS (n=604) | Severe PARDS (n=338) |
| --- | --- | --- | --- | --- |
| AVDSf<0.2  Lived  Died | a, b  1,531 (92.5%)  125 (7.5%) | c, d  722 (85.2%)  125 (14.8%) | f  291 (76.4%)  90 (23.6%) | g  81 (69.8%)  35 (30.2%) |
| AVDSf≥0.2 & <0.3  Lived  Died | a  88 (74.6%)  30 (25.4%) | c, e  94 (75.8%)  30 (24.2%) | 94 (70.7%)  39 (29.3%) | 57 (62.6%)  34 (37.4%) |
| AVDSf≥0.3  Lived  Died | b  36 (63.2%)  21 (36.8%) | d, e  36 (53.7%)  31 (46.3%) | f  50 (55.6%)  40 (44.4%) | g  61 (46.6%)  70 (53.4%) |
| p value | <0.0001 | <0.0001 | <0.0001 | 0.001 |

The first AVDSf within each PARDS category for each patient was used in the analysis. Some patients are represented multiple times across PARDS categories. There are no patients represented multiple times within a PARDS category. Reported p values are for across AVDSf category differences. For comparisons within each PARDS category, a p value of 0.0167 was considered significant using a Bonferroni correction. Significant differences between categories are represented by lower case letters.

**Supplement Table 2:** Model Performance for Mortality Multivariable Logistic Regression Models

| Model Time Window | n | Addition of AVDSf to Model with OI and PRISM | | | Addition of OI to Model with AVDSf and PRISM | | |
| --- | --- | --- | --- | --- | --- | --- | --- |
|  |  | LR Test (p value) | Decrease in AIC | Decrease in BIC | LR test (p value) | Decrease in AIC | Decrease in BIC |
| Intubation (0-6h) | 1181 | 0.017 | 3.68 | -1.39 | 0.041 | 2.17 | -2.90 |
| 12 hours (6-18h) | 1239 | 0.0005 | 10.15 | 5.03 | 0.19 | -0.28 | -5.40 |
| 24 hours (18-36h) | 1020 | 0.017 | 3.73 | -1.20 | 0.27 | -0.78 | -5.70 |
| 48 hours (36-60h) | 882 | 0.088 | 0.90 | -3.88 | 0.023 | 3.20 | -1.58 |
| 72 hours (60-84h) | 689 | 0.001 | 8.89 | 4.36 | 0.002 | 7.39 | 2.85 |
| 96 hours (84-120h) | 547 | 0.03 | 2.73 | -1.57 | 0.002 | 7.42 | 3.12 |
| 144 hours (120-168h) | 399 | 0.0003 | 11.30 | 7.31 | 0.001 | 8.27 | 4.28 |

AVDSf: end tidal alveolar dead space fraction. LR: Likelihood ratio test. AIC: BIC: OI: Oxygenation Index.

**Supplement Table 3:** Association between AVDSf and Mortality using Standardized Variables

| Model Time Window | n | AVDSf (per SD increase) | | Oxygenation Index (per SD increase) | | PRISM III Score (per SD increase) | |
| --- | --- | --- | --- | --- | --- | --- | --- |
|  |  | OR (95% CI) | p value | OR (95% CI) | p value | OR (95% CI) | p value |
| Intubation (0-6 h) | 1181 | 1.30 (1.05, 1.63) | 0.018 | 0.82 (0.67, 0.99) | 0.043 | 6.21 (4.74, 8.12) | <0.0001 |
| 12 hours (6 – 18h) | 1239 | 1.47 (1.18, 1.82) | <0.0001 | 0.89 (0.76, 1.06) | 0.19 | 5.45 (4.28, 6.94) | <0.0001 |
| 24 hours (18-36h) | 1020 | 1.29 (1.05, 1.58) | 0.017 | 1.11 (0.91, 1.35) | 0.27 | 4.75 (3.75, 6.04) | <0.0001 |
| 48 hours (36-60h) | 882 | 1.22 (0.97, 1.53) | 0.087 | 1.26 (1.03, 1.53) | 0.023 | 4.62 (3.59, 5.95) | <0.0001 |
| 72 hours (60-84h) | 689 | 1.55 (1.19, 2.00) | 0.001 | 1.42 (1.13, 1.79) | 0.003 | 4.62 (3.45, 6.20) | <0.0001 |
| 96 hours (84-120h) | 547 | 1.33 (1.03, 1.72) | 0.026 | 1.43 (1.13, 1.81) | 0.003 | 3.42 (2.57, 4.53) | <0.0001 |
| 144 hours (120-168h) | 399 | 1.82 (1.31, 2.53) | <0.0001 | 1.69 (1.20, 2.37) | 0.002 | 2.95 (2.14, 4.06) | <0.0001 |

Multivariable logistic regression models were constructed for each time window within the first 7 days of invasive mechanical ventilation using the identified AVDSf, Oxygenation Index, and PRISM III for that time window.

**Supplement Table 4:** Effect Estimates for AVDSf within each Age Category Considering Interaction between AVDSf and Age in Multivariable Mortality Models

| Model Time Window | n | Age Category | AVDSf (per 0.1 increase) | |
| --- | --- | --- | --- | --- |
|  |  |  | OR (95% CI) | p value |
| Intubation (0-6 h) | 1,181 | ≤1.2 years  >1.2 and <5.8 years  ≥5.8 and <13.2 years  ≥13.2 years | 0.91 (0.71, 1.16)  1.44 (1.04, 2.01)  1.43 (1.06, 1.94)  1.38 (1.01, 1.89) | 0.44  0.03  0.02  0.044 |
| 12 hours (6 – 18h) | 1,239 | ≤1.2 years  >1.2 and <5.8 years  ≥5.8 and <13.2 years  ≥13.2 years | 1.12 (0.83, 1.50)  2.04 (1.34, 3.11)  1.68 (1.12, 2.50)  1.45 (1.05, 2.01) | 0.47  0.001  0.12  0.025 |
| 24 hours (18-36h) | 1,020 | ≤1.2 years  >1.2 and <5.8 years  ≥5.8 and <13.2 years  ≥13.2 years | 1.08 (0.76, 1.53)  1.39 (1.00, 1.94)  1.26 (0.90, 1.76)  1.25 (0.92, 1.70) | 0.69  0.053  0.19  0.15 |
| 48 hours (36-60h) | 882 | ≤1.2 years  >1.2 and <5.8 years  ≥5.8 and <13.2 years  ≥13.2 years | 1.12 (0.78, 1.61)  1.19 (0.83, 1.71)  1.03 (0.70, 1.51)  1.54 (1.00, 2.36) | 0.55  0.34  0.88  0.048 |
| 72 hours (60-84h) | 689 | ≤1.2 years  >1.2 and <5.8 years  ≥5.8 and <13.2 years  ≥13.2 years | 1.00 (0.62, 1.61)  1.20 (0.80, 1.79)  1.60 (0.93, 2.73)  2.19 (1.45, 3.29) | 0.99  0.37  0.087  <0.0001 |
| 96 hours (84-120h) | 547 | ≤1.2 years  >1.2 and <5.8 years  ≥5.8 and <13.2 years  ≥13.2 years | 0.97 (0.55, 1.71)  0.86 (0.57, 1.29)  1.22 (0.70, 2.13)  1.84 (1.27, 2.67) | 0.92  0.48  0.49  0.001 |
| 144 hours (120-168h) | 399 | ≤1.2 years  >1.2 and <5.8 years  ≥5.8 and <13.2 years  ≥13.2 years | 1.48 (0.90, 2.45)  1.75 (1.00, 3.08)  1.31 (0.75, 2.27)  1.83 (1.19, 2.80) | 0.13  0.05  0.34  0.006 |

Multivariable logistic regression models adjust for Oxygenation Index and PRISM III. An interaction between Age and AVDSf was identified that was significant (p<0.2) for each time window model.

**Supplement Table 5:** Effect Estimates for AVDSf by Primary Diagnosis Category Considering Interaction between AVDSf and Primary Diagnosis in Mortality Models

| Model Time Window | n | Diagnosis Category | AVDSf (per 0.1 increase) | |
| --- | --- | --- | --- | --- |
|  |  |  | OR (95% CI) | p value |
| Intubation (0-6 h) | 1,181 | Pulmonary  Neurologic  Cardiovascular  Other | 0.96 (0.70, 1.30)  1.30 (0.95, 1.78)  1.22 (0.93, 1.59)  1.41 (1.02, 1.95) | 0.78  0.10  0.15  0.037 |
| 12 hours (6 – 18h) | 1,239 | Pulmonary  Neurologic  Cardiovascular  Other | 1.33 (0.94, 1.89)  1.84 (1.15, 2.92)  1.43 (1.00, 2.04)  1.19 (0.87, 1.63) | 0.11  0.011  0.051  0.28 |
| 24 hours (18-36h) | 1,020 | Pulmonary  Neurologic  Cardiovascular  Other | 1.14 (0.85, 1.53)  1.31 (0.83, 2.07)  1.31 (0.95, 1.83)  1.36 (0.94, 1.96) | 0.37  0.25  0.10  0.099 |
| 48 hours (36-60h) | 882 | Pulmonary  Neurologic  Cardiovascular  Other | 0.89 (0.59, 1.34)  1.54 (0.96, 2.46)  1.62 (1.08, 2.43)  0.98 (0.71, 1.37) | 0.58  0.075  0.019  0.91 |
| 72 hours (60-84h) | 689 | Pulmonary  Neurologic  Cardiovascular  Other | 1.30 (0.88, 1.90)  1.40 (0.75, 2.64)  1.58 (1.04, 2.39)  1.97 (1.20, 3.25) | 0.19  0.29  0.032  0.007 |
| 96 hours (84-120h) | 547 | Pulmonary  Neurologic  Cardiovascular  Other | 1.37 (1.00, 1.88)  0.96 (0.50, 1.85)  1.50 (0.92, 2.43)  1.18 (0.76, 1.81) | 0.05  0.90  0.10  0.47 |
| 144 hours (120-168h) | 399 | Pulmonary  Neurologic  Cardiovascular  Other | 2.12 (1.33, 3.37)  1.50 (0.77, 2.92)  2.01 (1.16, 3.48)  1.10 (0.68, 1.77) | 0.001  0.24  0.012  0.71 |

Multivariable logistic regression models adjust for Oxygenation Index and PRISM III. An interaction between Diagnosis Category and AVDSf was identified that was significant (p<0.2) for each time window model.

**Supplement Table 6:** Model Performance Length of Mechanical Ventilation in Survivors (Cox Regression) Models

| Model Time Window | n | Addition of AVDSf to Model with OI and PRISM III | | | Addition of OI to Model with AVDSf and PRISM III | | |
| --- | --- | --- | --- | --- | --- | --- | --- |
|  |  | LR Test (p value) | Decrease in AIC | Decrease in BIC | LR Test (p value) | Decrease in AIC | Decrease in BIC |
| Intubation | 1028 | 0.07 | 1.22 | -3.71 | <0.0001 | 15.76 | 10.83 |
| 12 hours (6-18h) | 1066 | <0.0001 | 22.92 | 17.95 | <0.0001 | 16.75 | 11.77 |
| 24 hours (18-36h) | 842 | 0.008 | 5.11 | 0.37 | 0.0001 | 13.84 | 9.11 |
| 48 hours (36-60h) | 725 | 0.001 | 8.26 | 3.68 | <0.0001 | 19.83 | 15.25 |
| 72 hours (60-84h) | 567 | 0.27 | -0.78 | -5.13 | <0.0001 | 17.24 | 12.90 |
| 96 hours (84-120h) | 442 | 0.009 | 4.83 | 0.74 | 0.002 | 8.12 | 4.03 |
| 144 hours (120-168h) | 308 | 0.17 | -0.09 | -3.82 | 0.001 | 8.36 | 4.63 |

AVDSf: end tidal alveolar dead space fraction. LR: Likelihood ratio test. AIC: BIC: OI: Oxygenation Index.

**Supplement Table 7:** Association between AVDSf and Time to Extubation in Survivors using Standardized Variables

| Model Time Window | n | AVDSf (per SD increase) | | Oxygenation Index (per SD increase) | | PRISM III Score (per SD increase) | |
| --- | --- | --- | --- | --- | --- | --- | --- |
|  |  | HR (95% CI) | p value | HR (95% CI) | p value | HR (95% CI) | p value |
| Intubation (0-6 h) | 1028 | 0.92 (0.85, 1.01) | 0.076 | 0.83 (0.76, 0.91) | <0.0001 | 0.81 (0.75, 0.88) | <0.0001 |
| 12 hours (6 – 18h) | 1066 | 0.81 (0.74, 0.88) | <0.0001 | 0.81 (0.73, 0.90) | <0.0001 | 0.89 (0.82, 0.96) | 0.002 |
| 24 hours (18-36h) | 842 | 0.89 (0.81, 0.97) | 0.009 | 0.80 (0.70, 0.90) | <0.0001 | 0.83 (0.76, 0.91) | <0.0001 |
| 48 hours (36-60h) | 725 | 0.86 (0.78, 0.95) | 0.002 | 0.77 (0.68, 0.86) | <0.0001 | 0.94 (0.86, 1.03) | 0.21 |
| 72 hours (60-84h) | 567 | 0.94 (0.84, 1.05) | 0.28 | 0.74 (0.65, 0.86) | <0.0001 | 0.85 (0.77, 0.94) | 0.002 |
| 96 hours (84-120h) | 442 | 0.84 (0.74, 0.96) | 0.011 | 0.80 (0.69, 0.93) | 0.003 | 0.97 (0.87, 1.07) | 0.51 |
| 144 hours (120-168h) | 308 | 0.89 (0.75, 1.06) | 0.18 | 0.73 (0.59, 0.90) | 0.003 | 0.98 (0.88, 1.10) | 0.75 |

Multivariable cox regression models were constructed for each time window within the first 7 days of invasive mechanical ventilation using the identified AVDSf, Oxygenation Index, and PRISM III for that time window.

**Supplemental Table 8:** Effect Estimates for AVDSf within each Age Category Considering Interaction between AVDSf and Age in Time to Extubation in Survivors Models

| Model Time Window | n | Age Category | AVDSf (per SD increase) | |
| --- | --- | --- | --- | --- |
|  |  |  | HR (95% CI) | p value |
| Intubation (0-6 h) | *No significant interaction between Age and AVDSf* | | | |
| 12 hours (6 – 18h) | 1065 | ≤1.2 years  >1.2 and <5.8 years  ≥5.8 and <13.2 years  ≥13.2 years | 0.92 (0.82, 1.02)  0.74 (0.62, 0.89)  0.85 (0.73, 1.00)  0.77 (0.67, 0.90) | 0.12  0.001  0.050  0.001 |
| 24 hours (18-36h) | 842 | ≤1.2 years  >1.2 and <5.8 years  ≥5.8 and <13.2 years  ≥13.2 years | 0.99 (0.87, 1.14)  0.90 (0.76, 1.05)  0.91 (0.78, 1.06)  0.82 (0.69, 0.96) | 0.94  0.18  0.22  0.015 |
| 48 hours (36-60h) | 725 | ≤1.2 years  >1.2 and <5.8 years  ≥5.8 and <13.2 years  ≥13.2 years | 0.90 (0.78, 1.02)  0.83 (0.70, 0.98)  0.97 (0.84, 1.12)  0.78 (0.64, 0.95) | 0.11  0.03  0.68  0.013 |
| 72 hours (60-84h) | 567 | ≤1.2 years  >1.2 and <5.8 years  ≥5.8 and <13.2 years  >13.2 years | 1.05 (0.91, 1.22)  0.90 (0.75, 1.08)  0.95 (0.76, 1.18)  0.83 (0.68, 1.03) | 0.51  0.27  0.65  0.09 |
| 96 hours (84-120h) | 442 | ≤1.2 years  >1.2 and <5.8 years  ≥5.8 and <13.2 years  >13.2 years | 1.06 (0.87, 1.30)  0.89 (0.74, 1.07)  0.65 (0.49, 0.86)  0.84 (0.69, 1.03) | 0.56  0.21  0.002  0.097 |
| 144 hours (120-168h) | 308 | ≤1.2 years  >1.2 and <5.8 years  ≥5.8 and <13.2 years  >13.2 years | 1.03 (0,83, 1.29)  0.90 (0.64, 1.26)  1.11 (0.84, 1.46)  0.70 (0.54, 0.91) | 0.75  0.52  0.46  0.007 |

Multivariable cox regression models adjust for Oxygenation Index, and PRISM III. An interaction between Age and AVDSf was identified that was significant (p<0.2) for each time window model except the Intubation model.

**Supplement Table 9:** Effect Estimates for AVDSf by Primary Diagnosis Category Considering Interaction between AVDSf and Primary Diagnosis in Time to Extubation in Survivors Models

| Model Time Window | n | Diagnosis Category | AVDSf (per SD increase) | |
| --- | --- | --- | --- | --- |
|  |  |  | HR (95% CI) | p value |
| Intubation (0-6 h) | 1,028 | Pulmonary  Neurologic  Cardiovascular  Other | 1.00 (0.91, 1.09)  0.89 (0.77, 1.02)  0.84 (0.71, 1.00)  0.99 (0.88, 1.10) | 0.97  0.10  0.056  0.83 |
| 12 hours (6 – 18h) | 1,066 | Pulmonary  Neurologic  Cardiovascular  Other | 0.95 (0.86, 1.06)  0.65 (0.53, 0.80)  0.91 (0.76, 1.09)  0.74 (0.65, 0.84) | 0.41  <0.0001  0.33  <0.0001 |
| 24 hours (18-36h) | 842 | Pulmonary  Neurologic  Cardiovascular  Other | 1.00 (0.90, 1.11)  0.76 (0.61, 0.95)  0.88 (0.72, 1.07)  0.84 (0.71, 0.98) | 0.95  0.016  0.20  0.025 |
| 48 hours (36-60h) | 725 | Pulmonary  Neurologic  Cardiovascular  Other | 0.92 (0.82, 1.03)  0.89 (0.71, 1.10)  0.83 (0.67, 1.03)  0.86 (0.73, 1.01) | 0.15  0.28  0.095  0.063 |
| 72 hours (60-84h) | 567 | Pulmonary  Neurologic  Cardiovascular  Other | 0.97 (0.85, 1.10)  0.84 (0.61, 1.16)  0.91 (0.74, 1.10)  0.99 (0.80, 1.24) | 0.60  0.30  0.33  0.95 |
| 96 hours (84-120h) | 442 | Pulmonary  Neurologic  Cardiovascular  Other | 1.03 (0.88, 1.20)  0.77 (0.57, 1.05)  0.81 (0.62, 1.06)  0.80 (0.64, 0.99) | 0.75  0.10  0.13  0.038 |
| 144 hours (120-168h) | 308 | Pulmonary  Neurologic  Cardiovascular  Other | 0.94 (0,74, 1.19)  1.05 (0.74, 1.48)  0.81 (0.58, 1.12)  0.84 (0.65, 1.09) | 0.60  0.79  0.19  0.19 |

Multivariable cox regression models adjust for Oxygenation Index and PRISM III. An interaction between Primary Diagnosis and AVDSf was identified that was significant (p<0.2) for each time window model.

**Supplement Table 10:** Association between AVDSf and Mortality limited to arterial blood gas data

| Model Time Window | n | AVDSf (per 0.1 increase) | | Oxygenation Index (per 1 increase) | | PRISM III | |
| --- | --- | --- | --- | --- | --- | --- | --- |
|  |  | OR (95% CI) | p value | OR (95% CI) | p value | OR (95% CI) | p value |
| Intubation (0-6 h) | 590 | 1.54 (1.19, 1.99) | 0.001 | 0.96 (0.93, 0.999) | 0.042 | 2.83 (2.28, 3.51) | <0.0001 |
| 12 hours (6 – 18h) | 810 | 1.65 (1.29, 2.11) | <0.0001 | 0.97 (0.95, 0.997) | 0.03 | 2.57 (2.19, 3.03) | <0.0001 |
| 24 hours (18-36h) | 717 | 1.44 (1.16, 1.78) | 0.001 | 0.996 (0.973, 1.02) | 0.73 | 2.29 (1.97, 2.65) | <0.0001 |
| 48 hours (36-60h) | 637 | 1.34 (1.06, 1.70) | 0.016 | 1.03 (0.99, 1.06) | 0.10 | 2.32 (1.97, 2.72) | <0.0001 |
| 72 hours (60-84h) | 501 | 1.85 (1.41, 2.43) | <0.0001 | 1.04 (0.995, 1.08) | 0.084 | 2.29 (1.91, 2.74) | <0.0001 |
| 96 hours (84-120h) | 414 | 1.38 (1.08, 1.73) | 0.010 | 1.05 (1.01, 1.09) | 0.014 | 1.90 (1.60, 2.25) | <0.0001 |
| 144 hours (120-168h) | 312 | 1.73 (1.28, 2.34) | <0.0001 | 1.07 (1.02, 1.12) | 0.008 | 1.82 (1.49, 2.22) | <0.0001 |

Multivariable logistic regression models were constructed for each time window within the first 7 days of invasive mechanical ventilation using the identified AVDSf, Oxygenation Index, and PRISM III for that time window.

**Supplement Table 11:** Association between AVDSf and Time to Extubation in Survivors limited to arterial blood gas data

| Model Time Window | n | AVDSf (per 0.1 increase) | | Oxygenation Index (per 1 increase) | | PRISM III | |
| --- | --- | --- | --- | --- | --- | --- | --- |
|  |  | HR (95% CI) | p value | HR (95% CI) | p value | HR (95% CI) | p value |
| Intubation (0-6 h) | 502 | 0.92 (0.82, 1.03) | 0.14 | 0.98 (0.96, 0.996) | 0.015 | 0.86 (0.81, 0.92) | <0.0001 |
| 12 hours (6 – 18h) | 664 | 0.79 (0.71, 0.89) | <0.0001 | 0.98 (0.96, 0.99) | 0.002 | 0.91 (0.86, 0.96) | <0.0001 |
| 24 hours (18-36h) | 562 | 0.88 (0.78, 0.98) | 0.017 | 0.98 (0.96, 0.99) | 0.003 | 0.87 (0.83, 0.92) | <0.0001 |
| 48 hours (36-60h) | 497 | 0.84 (0.75, 0.93) | 0.001 | 0.96 (0.94, 0.98) | <0.0001 | 0.97 (0.91, 1.02) | 0.22 |
| 72 hours (60-84h) | 388 | 0.85 (0.74, 0.97) | 0.018 | 0.96 (0.94, 0.99) | 0.003 | 0.89 (0.83, 0.95) | <0.0001 |
| 96 hours (84-120h) | 317 | 0.82 (0.71, 0.94) | 0.004 | 0.97 (0.95, 0.99) | 0.009 | 0.97 (0.91, 1.03) | 0.35 |
| 144 hours (120-168h) | 228 | 0.86 (0.72, 1.02) | 0.088 | 0.96 (0.93, 0.99) | 0.012 | 1.01 (0.93, 1.08) | 0.89 |

Multivariable cox regression models were constructed for each time window within the first 7 days of invasive mechanical ventilation using the identified AVDSf, Oxygenation Index, and PRISM III for that time window.
